# Dual fungal infection with *Lomentospora prolificans* and *Aspergillus fumigatus*: A pathogenetic diagnosis elucidated after two decades by sequential examinations

**DOI:** 10.1002/ccr3.3775

**Published:** 2021-01-18

**Authors:** Yoshitsugu Sugiura, Naoshi Sugimoto, Takayuki Takahashi, Tadahiko Matsumoto

**Affiliations:** ^1^ Azabu University Sagamihara Japan; ^2^ Department of Parasites Kobe Institute of Health Kobe Japan; ^3^ Department of Clinical Application Center for iPS Cell Research and Application Kyoto University Kyoto Japan; ^4^ Department of Hematology and Clinical Immunology Kobe City General Hospital Kobe Japan; ^5^ Department of Hematology and Oncology Akasaka Clinic Kobe Japan; ^6^ Ochanomizu Institute for Medical Mycology and Allergology Tokyo Japan; ^7^ Noguchi Dermatology Clinic Kumamoto Japan

**Keywords:** acute myeloid leukemia, aspergillosis, *Aspergillus fumigatus*, *Lomentospora prolificans*, orbital abscess, phaeohyphomycosis

## Abstract

A 44‐year‐old male Japanese was admitted for further post‐remission treatments for acute myeloid leukemia. He developed a right orbital abscess. An isolate of *Lomentospora prolificans* was obtained from the lesion, and orbital biopsy also revealed the presence of *Aspergillus fumigatus*. This fatal case involved a concurrent dual fungal infection.

## Graphical abstract

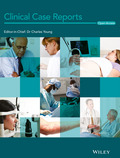

## 1 INTRODUCTION

Phaeohyphomycosis covers all infections caused by phaeoid fungi that do not fit into the classic concepts of chromoblastomycosis and black‐grained eumycotic mycetoma, which have muriform cells and granules as their respective hallmarks.^1^, ^2^

Members of the *Scedosporium apiospermum* species complex and *Lomentospora prolificans* (formerly *Scedosporium prolificans*) cause phaeohyphomycosis and may include asymptomatic colonization, localized infection, and disseminated infection. They act as primary or opportunistic pathogens in immunocompetent and immunocompromised hosts.^3^

Three species are considered medically important, namely *S. apiospermum*, *S. boydii*, and *L. prolificans*. These fungi are frequently isolated from the respiratory secretions of patients suffering from chronic pulmonary conditions such as cystic fibrosis.^4^ *Lomentospora prolificans* was shown to be unrelated to *Scedosporium* and reclassified as *L*. *prolificans,* and the genus *Lomentospora* was reinstated for this species.^5^

When multifocal fungal infections are found in a highly immunocompromised patient, physicians should be prepared for possible double or more fungal pathogens rather than single one, especially in the case that the effect of antifungal agents is insufficient on the lesions. We herein describe an uncommon case of dual fungal infection, including *L*. *prolificans*, in a leukemic patient.

## 2 CASE REPORT

The patient was a 44‐year‐old Japanese man who developed acute myeloid leukemia (AML) and who was admitted to Kobe City General Hospital in July 2000 (Day 0). Remission was achieved with chemotherapy. Subsequently, consolidation therapy and intensive maintenance therapy were repeated. In May 2001 (Month + 10), he was hospitalized for a second intensive maintenance therapy session. Computed tomography and magnetic resonance imaging revealed systemic lesions in the brain, right orbit, lungs, spine, and liver during the period of neutropenia. After recovery of the neutrophil count, the right orbital abscess enlarged and compressed the optic nerve. It was surgically removed in June 2001 (Month + 11) due to pain and reduced vision. The resected specimen included the abscess and surrounding bone. The abscess was surrounded by inflammatory granulomatous tissue, and the orbital bone showed black pigmentation, suggesting a phaeoid fungal infection.

**FIGURE 1:**
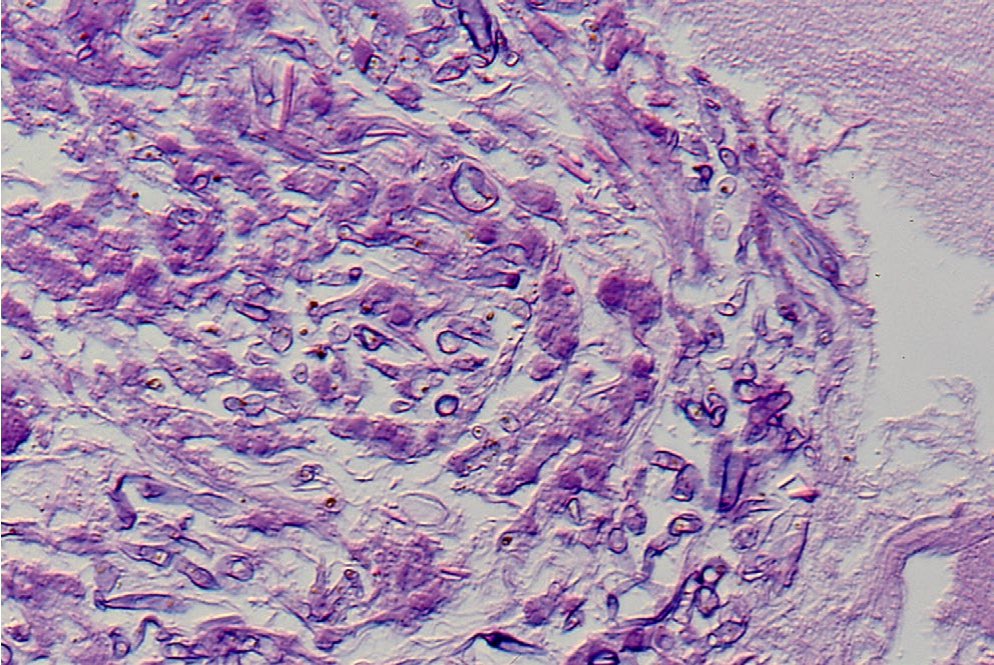
Aspergillosis of the orbital specimen (H&E staining) × 200

**FIGURE 2:**
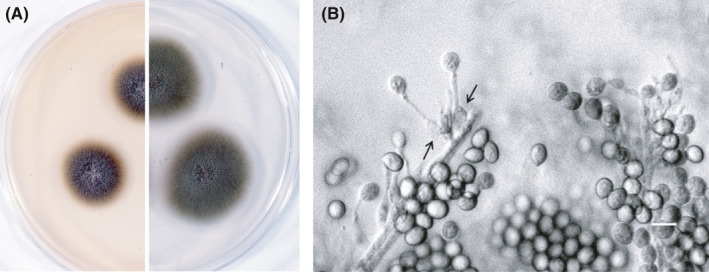
Morphology of *Lomentospora prolificans*. A, The colony size was 17‒18 mm and 22‒23 mm on modified Sabouraud dextrose agar (left) supplemented with 1.5% potato dextrose agar plus 0.3% malt extract and potato dextrose agar (right), respectively, B, Flask‐shaped conidiogenous cells (arrow) and a long neck (bar, 10 µm)

As the first choice of chemotherapy, fluconazole was administered in combination with antibiotics; however, the fever continued, and the C‐reactive protein (CRP) level elevated. A pathological examination revealed aspergillosis based on H&E staining of the orbital tissues (Figure 1), but it could not identify the *Aspergillus* species. Later, a molecular diagnosis based on the sequence of the internal transcribed spacer 1 (ITS1) region of the ribosomal RNA gene using a preserved pathological specimen revealed that the causative species was *A. fumigatus* (234/236 bases). In the smear culture of the orbital specimen, a slow‐growing colony was velvety and olive to dark gray in color (Figure 2). The conidia were ovoid to subglobose, smooth‐walled, olive to brown, and 7‒8 × 3‒5 μm in size. The fungus was morphologically identified as “*Scedosporium prolificans,”* in compliance with the taxonomy at that time. At this stage, it became clear that the patient had a dual fungal infection. The minimum inhibitory concentrations for *L. prolificans* were as follows: amphotericin B, 4 μg/mL; 5‐fluorocytocine (5‐FC), >64 μg/mL; miconazole, 4‒8 μg/mL; fluconazole, >64 μg/mL; and itraconazole, >8 μg/mL. The sequence of the ITS 1 region of the *S. prolificans* isolate was identical to the sequence of *L. prolificans*, according to the previous method.^6^

Amphotericin B was administered based on the pathological diagnosis of aspergillosis. The fever and the CRP level gradually decreased. However, the spinal lesions did not decrease, and the CRP level remained > 10 mg/mL. Miconazole was temporarily used for the spinal lesions but was ineffective. Combined therapy with amphotericin B and 5‐FC briefly stabilized the clinical status. His leukemia relapsed in September 2001 (Month + 14) and was treated with another series of chemotherapy. The patient's symptoms worsened, and he eventually died of sudden respiratory arrest in October 2001 (Month + 15).

## 3 DISCUSSION

Phaeohyphomycosis and hyalohyphomycosis were introduced as umbrella terms to cover a growing number of opportunistic mycoses regardless of the site of the lesion, the pattern of tissue response, granuloma, or abscess, or the taxonomic classification of the etiologic agents caused by moulds and yeasts, whose septate mycelial tissue forms were either phaeoid (Gr “phaios” = dusky, melanin‐pigmented) or hyaline (Gr “hyaleos” = non‐pigmented), respectively. Phaeohyphomycosis caused by *L. prolificans* has increased as cases of disseminated infection in immunocompromised patients.^7^ Several fatal cases have been reported in France,^8^ Spain,^9^, ^10^, ^11^ Australia,^12^, ^13^ and the United States.^3^, ^7^ However, disseminated *L. prolificans* infection is uncommon in Japan ^14^, ^15^, ^16^, ^17^ and Thailand.^18^

*Lomentospora prolificans* is well known to be resistant against several antifungal drugs.^10^, ^11^ Therefore, it is difficult to cure *L. prolificans* infection, and the early diagnosis of these patients is required to start antifungal treatment. In the present case, the isolate was resistant to 5‐FC and fluconazole, and less resistant to amphotericin B, miconazole, and itraconazole. This *L. prolificans* isolate has been deposited with the strain number IFM 51110 at the Research Center for Pathogenic Fungi and Microbial Toxicoses, Chiba University. The strain is maintained through the National Bio‐Resource Project (NBRP), Japan.

In this patient, fungal infections were found as systemic lesions, such as in the brain, right orbit, lungs, spine, and liver. Right orbital bone blackening was caused by infection with the phaeoid fungus, *L. polificans*. After the diagnosis of aspergillosis, a combination of amphotericin B and 5‐FC was prescribed, and these drugs were effective, suggesting that the *A. fumigatus* in the patient's lungs was susceptible to this antifungal treatment. However, the spinal lesions did not respond to fluconazole, miconazole, amphotericin B, or 5‐FC, and his CRP level remained > 10 mg/mL. Invasive infections of *A. fumigatus* in humans are known to occur in the lungs, nasal sinuses, and the brain^19^; however, no bone infections have been reported. On the other hand, *L. prolificans* was initially reported to cause bone and joint infections,^7^ thus suggesting that the spinal lesions may be caused by *L. prolificans* infection.

Since no autopsy was performed due to the confirmed diagnosis of AML and aspergillosis, it remains unknown which lesions were caused by *A. fumigatus* or *L. prolificans* infection. Dual infection with *L. prolificans* and *A. fumigatus* had not been reported in leukemic patients in Japan, and *L. prolificans* was not considered to be an emerging fungal pathogen among medical scientists. However, an autopsy should have been performed to identify the causative agent of the spinal lesions. The diagnosis of this dual infection became possible after two decades when the sequential examination was established. Thus, we report this instructive clinical case that was hidden in the past.

## 4 CONCLUSION

Fungal infections that appear in immunosuppressed patients show various symptoms, sites of lesions, and pathological responses. Our experience taught us a bitter and precious lesson. We should consider the possibility that multiple pathogenic microorganisms can reside in a critical medical situation.

## CONFLICT OF INTEREST

The authors declare no conflict of interest in association with the present study.

## AUTHOR CONTRIBUTIONS

YS: (corresponding author) contributed to this manuscript by identifying fungi, studying the antifungal susceptibility, and collecting information for the case report. TM: contributed to this manuscript by researching other cases of phaeohyphomycosis after drafting the discussion. NS and TT: contributed to the patient's care; initially diagnosed the patient; and performed the follow‐up care. All authors: reviewed and approved the final manuscript.

## ETHICAL APPROVAL

The consent could not be obtained because the patient deceased 19 years ago. Unfortunately, the authors could not trace his relatives, either. Therefore, we have carefully anonymized the information so that the patient cannot be identified. The authors declare human ethics approval was not needed for this study.

## Data Availability

Data are openly available in a public repository that issues dataset with DOIs.
